# miR-181b/Notch2 overcome chemoresistance by regulating cancer stem cell-like properties in NSCLC

**DOI:** 10.1186/s13287-018-1072-1

**Published:** 2018-11-23

**Authors:** Xiaoyuan Wang, Qingwei Meng, Wenbo Qiao, Ruishuang Ma, Weiwei Ju, Jing Hu, Hailing Lu, Jianqi Cui, Zhao Jin, Yanbin Zhao, Yan Wang

**Affiliations:** 1 The Department of Internal Medical Oncology, Harbin Medical University Cancer Hospital, Harbin, Heilongjiang Province China; 2 The Department of Radiotherapy, Harbin Medical University Cancer Hospital, Harbin, Heilongjiang Province China; 3 Pathology Department, Laboratory of Molecular Medicine, College of Medicine, Eastern Liaodong University, Dandong, Liaoning Province China

**Keywords:** MicroRNA-181b, Non-small cell lung cancer (NSCLC), Notch2, Chemosensitivity, Cancer stem cell-like properties

## Abstract

**Background:**

Lung cancer stem cells have the ability to self-renew and are resistant to conventional chemotherapy. MicroRNAs (miRNAs) regulate and control the expression and function of many target genes; therefore, miRNA disorders are involved in the pathogenesis of human diseases, such as cancer. However, the effects of miRNA dysregulation on tumour stemness and drug resistance have not been fully elucidated. miR-181b has been reported to be a tumour suppressor miRNA and is associated with drug-resistant non-small cell lung cancer.

**Methods:**

Cancer stem cell (CSC)-like properties were tested by a cell proliferation assay and flow cytometry; miR-181b expression was measured by real-time PCR; and Notch2 and related proteins were detected by Western blotting and immunohistochemistry. A mouse xenograft model was also established.

**Results:**

In this study, we found that ectopic miR-181b expression suppressed cancer stem cell properties and enhanced sensitivity to cisplatin (DDP) treatment by directly targeting Notch2. miR-181b could inactivate the Notch2/Hes1 signalling pathway. In addition, tumours from nude mice treated with miR-181b were significantly smaller than tumours from mice treated with control agomir. Decreased miR-181b expression and increased Notch2 expression were observed to have a significant relationship with overall survival (OS) and CSC-like properties in non-small cell lung cancer (NSCLC) patients.

**Conclusions:**

This study elucidates an important role of miR-181b in the regulation of CSC-like properties, suggesting a potential therapeutic target for overcoming drug resistance in NSCLC.

**Electronic supplementary material:**

The online version of this article (10.1186/s13287-018-1072-1) contains supplementary material, which is available to authorized users.

## Background

Lung cancer is the leading cause of cancer deaths worldwide. Non-small cell lung cancer (NSCLC) accounts for approximately 85% of all lung cancer cases [1, 2]. Approximately 70% of patients present with locally advanced or metastatic disease at diagnosis. Despite improvements in the diagnosis and treatment of lung cancer, the 5-year survival rate remains low (< 15%), largely due to the emergence of resistance before and during the course of treatment with chemotherapy and radiation therapy [3, 4]. This resistance to therapy represents a significant clinical challenge in the treatment of lung cancer and contributes greatly to disease progression, recurrence, and mortality.

Despite intense efforts to develop novel therapeutics as single agents and in combination with chemoradiotherapy, the mechanisms underlying this resistant phenotype in lung cancer are largely unclear [5]. Cancer stem cells (CSCs) are currently recognized as a unique subset of cells with exclusive abilities such as unlimited self-renewal, asymmetric cell division, and resistance to toxic agents that enable them to perpetuate the growth of a malignant population, which distinguishes them from the bulk of tumour cells [6]. These important clinical observations of CSC-like properties have triggered intense interest in experimental approaches to further investigate their effects on the treatment of drug-resistant lung cancer.

MicroRNAs (miRNAs), small noncoding RNA molecules that suppress gene expression by interacting with the 3′ untranslated regions (3′-UTRs) of target messenger RNAs, regulate many biological activities, such as cancer metastasis and chemosensitivity, and influence CSCs [7–9]. It has been shown that microRNA-181b (miR-181b) is one of the most markedly downregulated miRNAs in human NSCLC compared with normal tissues [10]. Importantly, low miR-181b expression promotes proliferation and predicts cisplatin chemoresistance in NSCLC [11, 12].

A recent study reported that miR-181b is associated with temozolomide resistance in U87 glioma stem cells [13]. These results suggest that miR-181b might be involved in chemosensitivity by regulating stem cell-like properties in cancer. However, the functional role of miR-181b in NSCLC, especially in regulating CSC-like properties, remains largely unknown.

In our studies, we showed that miR-181b regulates chemosensitivity by suppressing the stem-like characteristics of NSCLC in vitro and in vivo. Notch2 is a target gene of miR-181b that suppresses Notch2 signalling to inhibit CSC traits. In addition, the expression of miR-181b and Notch2 was significantly related to stemness and prognosis in patients with NSCLC. This study elucidates an important role of miR-181b in the regulation of CSC-like properties, suggesting that the miR-181b-Notch2 axis is a potential target for the treatment of chemoresistance in NSCLC.

## Methods

### Cell lines and tumoursphere cultures

The NSCLC cell lines H1650, H1299, A549, and A549/DDP were obtained from Heilongjiang Cancer Institute (Harbin, China). Cells were cultured in RPMI-1640 medium supplemented with 10% foetal bovine serum, 50 U/mL penicillin, and 50 mg/mL streptomycin at 37 °C in a humidified atmosphere containing 5% CO_2_. To maintain the multidrug resistance (MDR) phenotype, DDP (1 μg/mL) was added to the culture media for A549/DDP cells. For tumoursphere cultures, cells (1000 cells/mL) were cultured in suspension in serum-free DMEM/F12 supplemented with 1% penicillin, B27 (1:50, Gibco), 20 ng/mL epidermal growth factor (Prospec), 5 mg/mL insulin (Sigma), and 0.4% BSA (Sigma). After 10 to 12 days of culture, the plates were analysed for tumoursphere formation, which was quantified using a microscope (Olympus).

### Cell proliferation assay

Suspensions of NSCLC cell lines (1 × 10^5^/well) were plated in 96-well plates and grown at 37 °C with different concentrations of DDP. Then, the cells were treated with DDP for 24 h. After treatment, the media were removed, and RPMI1640 (90 μL) and CCK-8 (10 μL) were added. The plates were incubated for 3 h in the incubator. The absorbance at 450 nm was measured on an automated reader. The inhibition rate (IR) was calculated.

$$ \mathrm{IR}=\left({\mathrm{OD}}_{\mathrm{control}\ \mathrm{group}}-{\mathrm{OD}}_{\mathrm{experimental}\ \mathrm{group}}\right)/{\mathrm{OD}}_{\mathrm{control}\ \mathrm{group}}\times 100\%. $$

### Real-time PCR

Total cellular RNA was isolated from cultured cells using TRIzol reagent (Invitrogen). RNA samples (500 ng each) were then reverse-transcribed into cDNA with miR-181b reverse transcriptase and primers using a TaqMan MicroRNA Reverse Transcription Kit (Applied Biosystems). Levels of miR-181b and U6 expression were determined by qPCR with TaqMan MicroRNA Assays and an ABI 7500 instrument (Applied Biosystems). The levels of mature miR-181b expression were then normalized to U6 and calculated as the inverse log of the ΔCt (relative mRNA abundance was calculated as 2^−ΔCt [ΔCt = Ct (miR–181b) − Ct(U6)]^). All procedures were performed following the manufacturer’s instructions.

### miRNA mimics and inhibitors and gene transfection

The cells were cultured in 6-well plates to 40% confluence. miR-181b mimics, miR-181b inhibitors, or the negative control Notch2 or si-Notch2 (Sigma) was mixed with Lipofectamine 2000 (Invitrogen) and then added to the cell culture medium according to the manufacturer’s instructions. After 24 h of transfection, total RNA and protein were extracted from cells and subjected to qRT-PCR and Western blot analyses, respectively.

### Flow cytometry for apoptosis analysis and surface marker analysis of CD133

Cells were incubated with culture medium containing DDP for 24 h after transfection with miR-181b mimics, miR-181b inhibitor, or negative control. For apoptosis analysis, cells were then collected, washed with PBS, resuspended in 100 μL of 1× binding buffer and stained with 5 μL of Annexin V and 5 μL of PI (Becton- Biosciences) at room temperature for 15 min in the dark. A flow cytometer (Becton Biosciences) was utilized to evaluate the levels of apoptosis in each sample following the manufacturer’s instructions.

We assessed the expression of the CD133 surface marker after transfection with miR-181b mimics (Invitrogen) or Notch2 siRNA (Santa Cruz Biotechnology). Briefly, cells were washed with PBS, stained with anti-CD133 (PE-conjugated; Miltenyi Biotec) antibody in PBS containing 1% FBS and incubated on ice in the dark for 30 min. The cells were washed again with cold PBS, and > 10,000 cells were analysed by flow cytometry. The data were analysed using FACS Diva software.

### Dual luciferase reporter assay

Cells (1 × 10^5^ H1299/well) were cultured in 24-well plates and transfected with Notch2–3′UTR-wt or Notch2–3′-UTR-mt and miR-181b or miR-NC using Lipofectamine 2000 (Invitrogen, USA) according to the manufacturer’s protocol. Luciferase activity was measured 24 h after transfection using the Dual Luciferase Reporter Assay System (Promega) and normalized to Renilla luciferase activity.

### Immunohistochemistry

Each patient signed an informed consent form for medical record review and tissue sample donation. This study was approved by the Institutional Review Board at Harbin Medical University and was conducted according to all current ethics guidelines. Tissue sections were immersed in MEDTA, incubated in a steam bath at 100 °C for 15 min, and incubated in methanol containing 0.3% H_2_O_2_ for 15 min. The slides were incubated with Notch2 (1:200 dilution, Abclonal), CD133 (1:200 dilution, JKSJ-orb372326), and SOX2 (1:100 dilution, Abcam-ab92494) primary antibodies, stained using DAB, and counterstained using haematoxylin. The staining results were independently interpreted by two pathologists in a blinded manner. For each slide, three to five randomly selected fields were evaluated. For each field, the percentage of DAB-positive tumour cells was calculated as [(number of DAB-positive tumour cells/total number of tumour cells) × 100]. The relative staining intensity was defined as negative for 5%, weak (+) for 5–25%, moderate (++) for 25–50%, and strong (+++) for 50% of tumour cells stained positive for Notch2.

### In vivo

All animal experiments were undertaken in accordance with the NIH Guide for the Care and Use of Laboratory Animals with the approval of the Scientific Investigation Board of the Affiliated Tumor Hospital of Harbin Medical University. BALB/c athymic nude mice (male, 4–6 weeks old) were purchased from the Shanghai Experimental Animal Center of the Chinese Academy of Sciences (Shanghai, P.R. China) and bred under pathogen-free conditions in the Animal Center of the Affiliated Tumor Hospital of Harbin Medical University. A total of 1 × 10^4^, 1 × 10^5^, or 1 × 10^6^ A549/DDP cells were injected subcutaneously into the left flank of each mouse. After 8 days, the transplanted nude mice were randomly divided into three groups (*n* = 6 each). Agomir-miR-181b or agomir-NC (RiboBio Co., Ltd., Guangzhou, China) was directly injected into the implanted tumour at a dose of 1 nmol per mouse every 4 days for seven total injections. Tumour volume (*V*) was monitored by measuring the length (*L*) and width (*W*) and calculated by the formula *V* = (*L* × *W*^2^) × 0.5. To evaluate whether the miR-181b level was associated with NSCLC sensitivity to DDP, mice began receiving DDP treatment when the mean tumour volume reached 200 mm^3^. Upon termination, each mouse was weighed, and tumours were harvested for immunohistochemistry analysis, Western blot analysis, and qPCR. The immunohistochemistry analysis was performed according to our previously described method. Four or 7 weeks later, the mice were sacrificed, and the tumours were harvested and fixed in formalin. The fixed samples were then embedded in paraffin, and the sections were stained with antibody.

### Statistical analysis

The results are presented as the mean ± SD of three independent experiments. Statistical differences were evaluated by Student’s *t* test or ANOVA as appropriate. Survival curves were analysed by the Kaplan–Meier method. The criterion for statistical significance was set at *p* < 0.05. Statistical analysis was performed using SPSS 18.0.

## Results

### Restoration of miR-181b expression reduces cancer stem cell-like properties in NSCLC

CSCs are considered an important factor for tumour initiation, drug resistance, and cancer recurrence. Previous studies have demonstrated that some miRNAs regulate the functions of CSCs. We have previously confirmed that miR-181b regulates chemosensitivity in NSCLC [36]. To confirm the role of miR-181b in regulating CSC-like properties such as chemoresistance, a tumoursphere formation assay was utilized to test the effects of miR-181b restoration on the sphere formation of NSCLC cells. Initially, significantly lower expression of miR-181b was observed in A549/DDP and H460 cells than in A549 and H1650 cells, which have lower expression levels than HBE normal lung epithelial cells, as detected by qPCR (Additional file 1: Figure S1A).

**Fig. 1:**
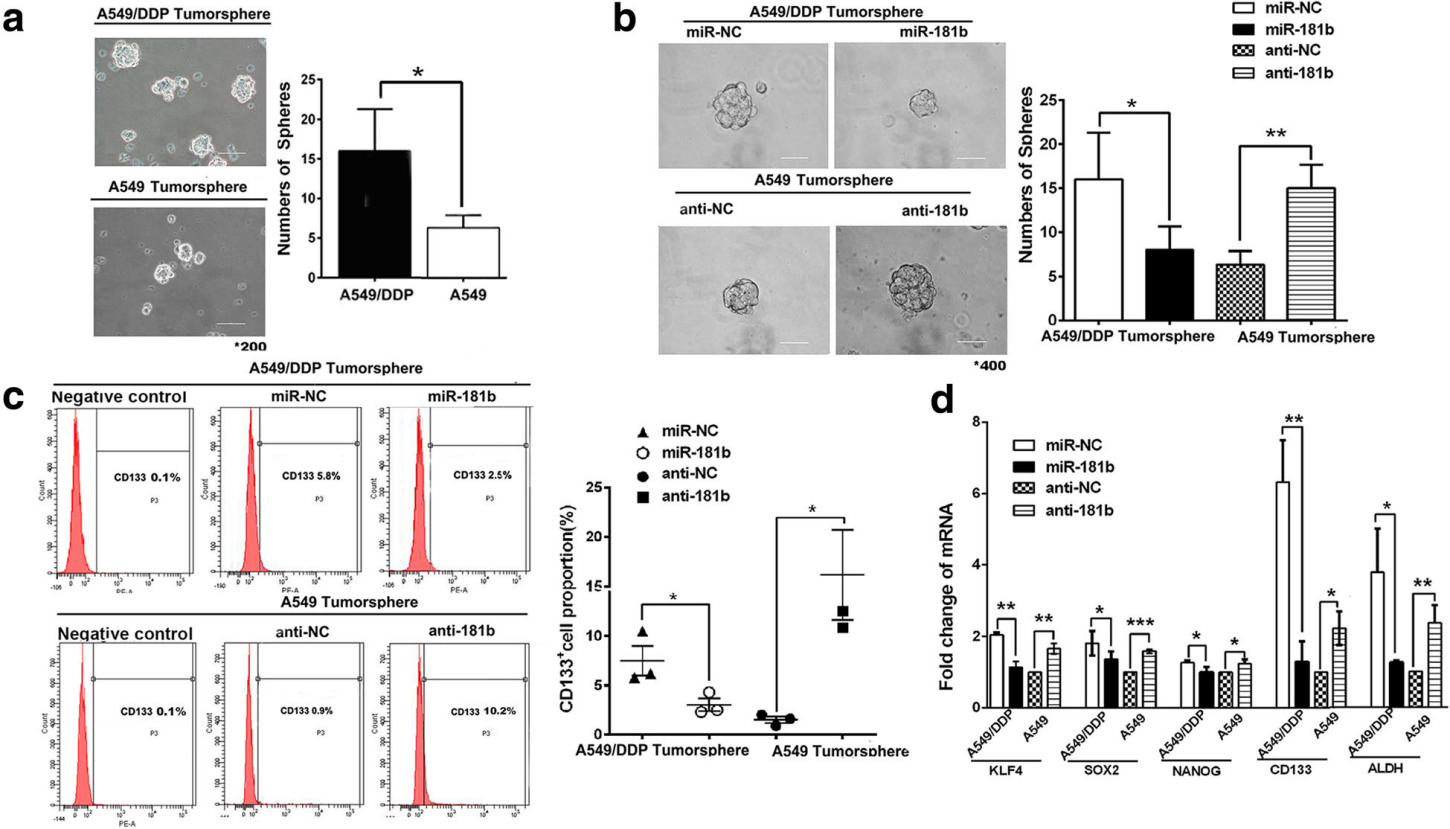
Reduced miR-181b expression promotes CSC properties in NSCLC. A549/DDP and A549 cells were transfected with miR-181b mimics, miR-181b inhibitors, or the control. **a**, **b** The number of tumourspheres were counted, and the morphology was observed under a light microscope. **c** CD133^+^ cells were analysed in A549/DDP and A549 spheres by flow cytometry. **d** The mRNA levels of KLF4, SOX2, NANOG, CD133, and ALDH were measured by qPCR. Bars represent 200 μm for the low-power lens and 50 μm for the high-power lens. Data are presented as the mean ± SD. *p < 0.05; **p < 0.01; ***p < 0.001

After being cultured in serum-free media with growth factors, A549/DDP, H460, A549, and H1650 cells lost the capacity to adhere to the plate and clustered in multicellular spheroids. A549/DDP cells formed more and larger tumourspheres than did A549 cells (*p* < 0.05, Fig. 1a). Cells with miR-181b formed fewer and smaller tumourspheres than did cells transfected with the negative control. More and larger tumourspheres formed following anti-miR-181b exposure in A549 cells (*p* < 0.05 and *p* < 0.01, Fig. 1b). To determine whether spheres were enriched in CSCs, several putative CSC markers were analysed. Cells positive for CD133 (a marker of LCSCs) display self-renewal and tumour-initiating abilities in NSCLC [14, 15]. A549/DDP spheres contained more cells highly expressing CD133 than did A549 spheres. Overexpression of miR-181b reduced the CD133^+^ population following transfection with miR-181b mimics, whereas the miR-181b inhibitor augmented the CD133^+^ population (Fig. 1c). Moreover, stemness-related genes such as KLF4, SOX2, NANOG, CD133, and ALDH were upregulated in A549/DDP sphere cells compared to A549 sphere cells. These genes were also downregulated in miR-181b mimic-transfected cells compared to NC cells, whereas the miR-181b inhibitor increased the expression of KLF4, SOX2, NANOG, CD133, and ALDH, as shown by qPCR (Fig. 1d). The same results were found in H460 and H1650 cells (Additional file 1: Figure S1B-D). Taken together, our results suggest that miR-181b suppresses the stem-like activities of NSCLC cells.

### Ectopic miR-181b expression enhances the sensitivity to DDP

**Fig. 2:**
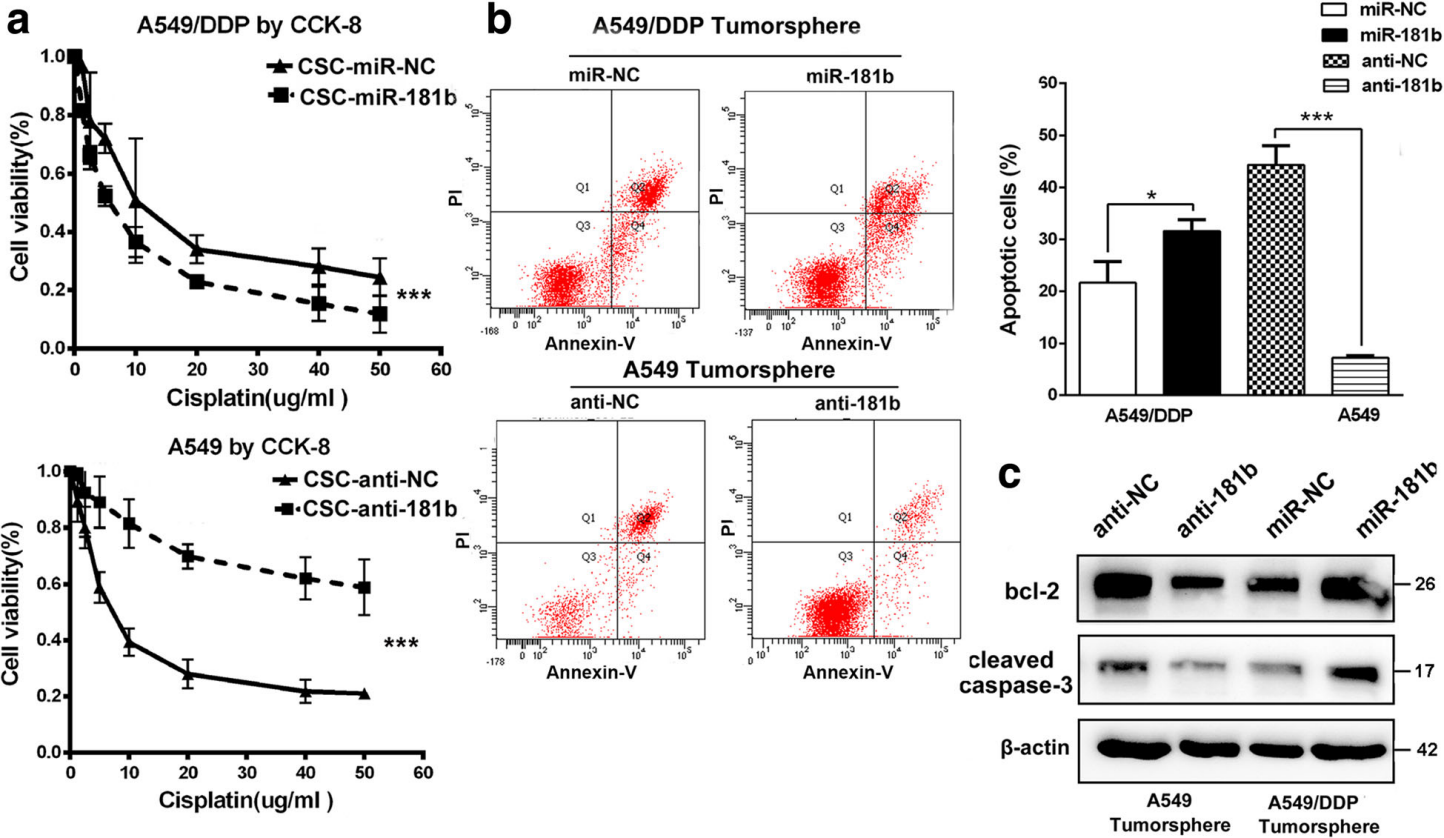
miR-181b enhances the sensitivity of NSCLC cells to DDP. After transfection with miR-181b mimics, miR-181b inhibitors, or the control, A549/DDP and A549 spheres were analysed. **a** IC50 values by CCK analysis with different concentrations of cisplatin, **b** apoptotic percentage by flow cytometry, and **c** Bcl-2 and cleaved caspase-3 expression by Western blotting. Data are presented as the mean ± SD. *p < 0.05; **p < 0.01; ***p < 0.001

Drug resistance is an important issue in cancer patients undergoing chemotherapy. CSCs are naturally resistant to chemotherapy, enabling them to survive anticancer drugs and support the re-growth of other cancer cell populations [16, 17]. Therefore, we examined whether miR-181b expression affects DDP resistance in cells within spheres using the CCK assay. As expected, A549/DDP spheres with miR-181b were more sensitive than miR-NC spheres to DDP treatment, while A549 spheres with anti-miR-181b were more resistant than miR-NC spheres to DDP treatment (Fig. 2a). Then, to determine the percentage of Annexin-FITC/PI double-positive cells, FACS analysis was performed. After transfection with miR-181b mimics, the percentage of apoptotic cells increased, suggesting that miR-181b mimics re-sensitized A549/DDP spheres to DDP. When miR-181b expression was downregulated, the percentage of apoptotic cells in A549 spheres decreased (Fig. 2b). Moreover, miR-181b increased the expression of Bcl-2 and cleaved caspase-3 levels in A549/DDP spheres, while anti-miR-181b decreased the expression of Bcl-2 and cleaved caspase-3 levels in A549 spheres following treatment with DDP (Fig. 2c). These results were also confirmed in H460 and H1650 cells (Additional file 2: Figure S2A-D). Collectively, miR-181b enhances the sensitivity of NSCLC sphere cells to DDP, resulting in apoptosis.

### miR-181b suppresses Notch2 signalling to inhibit CSC traits

**Fig. 3:**
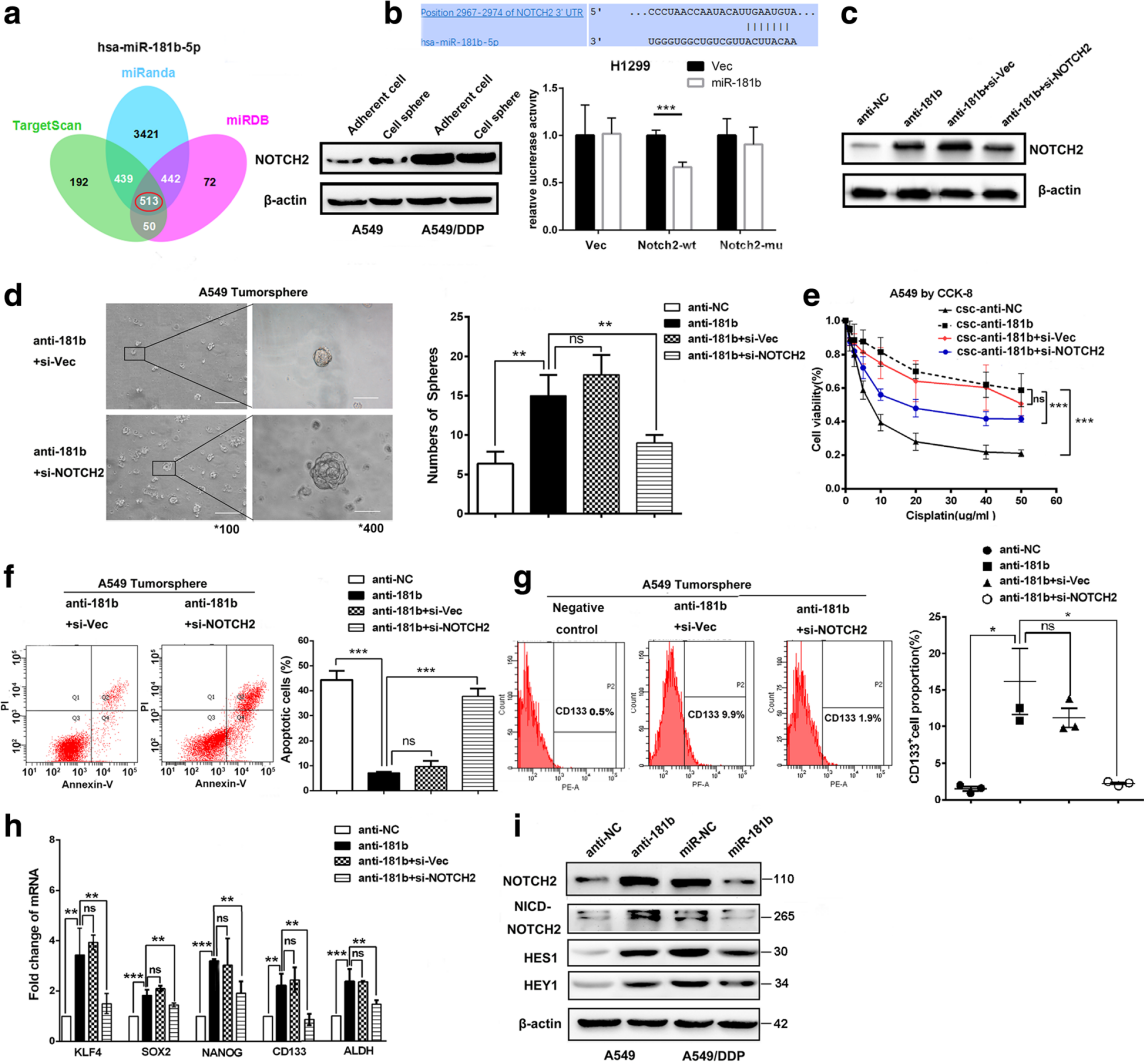
miR-181b suppresses Notch2 signalling to inhibit CSC traits. **a** Venn diagrams show the number of genes identified as potential targets of miR-181b in three predictive programs: TargetScan, miRanda, and miRBD. Notch2 expression in adherent A549/DDP and A549 cells or cell spheres was tested by Western blotting. **b** Relative luciferase activity was evaluated after wild-type or mutant 3′-UTR reporter plasmids were co-transfected with pGLE-miR-181b or miR-181b-NC in H1299 cells. **c** A549 cells were transfected with miR-181b inhibitors or the control and then treated with si-Notch2 or negative control. Notch2 expression was analysed by Western blotting. **d** The number of tumourspheres was counted, and the morphology was observed under a light microscope. **e** The IC50 value was determined by CCK assay with different concentrations of cisplatin. The apoptotic percentage (**f**) and CD133^+^ cells (**g**) were evaluated by flow cytometry. **h** The mRNA levels of KLF4, SOX2, NANOG, CD133, and ALDH were measured by qPCR. **i** Western blotting showed Notch2, NICD2, HES1, and HEY1 expression after A549/DDP and A549 cells were transfected with miR-181b mimics, miR-181b inhibitors, or the control. Bars represent 200 μm for low-power lens and 50 μm for high-power lens. Data are presented as the mean ± SD. *p < 0.05; **p < 0.01; ***p < 0.001

Having established a link between miR-181b and CSC traits, we further investigated the mechanism of miR-181b by identifying its downstream targets. We employed the TargetScan, miRanda, and miRDB programs to predict potential target genes. Notch2, a stem cell gene related to mammary stem cells and carcinogenesis, piqued our interest when it was identified as a potential target of miR-181b. To test this hypothesis, we analysed the expression of Notch2 in NSCLC cell lines. As expected, Notch2 expression was markedly higher in A549/DDP cells than in A549 cells (Fig. 3a).

To further evaluate whether miR-181b affects Notch2 expression, H1299 cells were transfected with miR-181b mimics or scrambled miRNA (negative control). The luciferase constructs cloned into the pGL3-control vector were used to examine the ability of miR-181b to target the 3′-UTR of Notch2 mRNA. The luciferase activity of the wild-type Notch 2 3′-UTR was reduced following ectopic miR-181b expression, but this reduction was not observed with mutant constructs (*p <* 0.001, Fig. 3b).

Next, the role of Notch2 activation in miR-181b-induced stemness was further examined. Figure 3d, e, and f show that silencing Notch2 in anti-miR-181b-A549 cells strikingly reduced the ability of NSCLC cells to form spheres in vitro and increased the sensitivity to DDP. FACS analysis indicated that the downregulation of Notch2 also reduced the CD133^+^ population (Fig. 3g). In addition, stemness-related transcription factors (KLF4, SOX2, NANOG, CD133, and ALDH) were downregulated in Notch2 siRNA cells (Fig. 3h). Notch receptors signal through the transcriptional activation of the target genes HES-1 and Hey1. We found that miR-181b mimics significantly downregulated Notch2, NICD2, HES1, and HEY1 expression, while anti-miR-181b significantly upregulated the expression of these genes (Fig. 3i). These data indicate that Notch2 plays a critical role in the miR-181b-mediated stemness of NSCLC cells.

### miR-181b regulates cancer stem cell-like characteristics in vivo

**Fig. 4:**
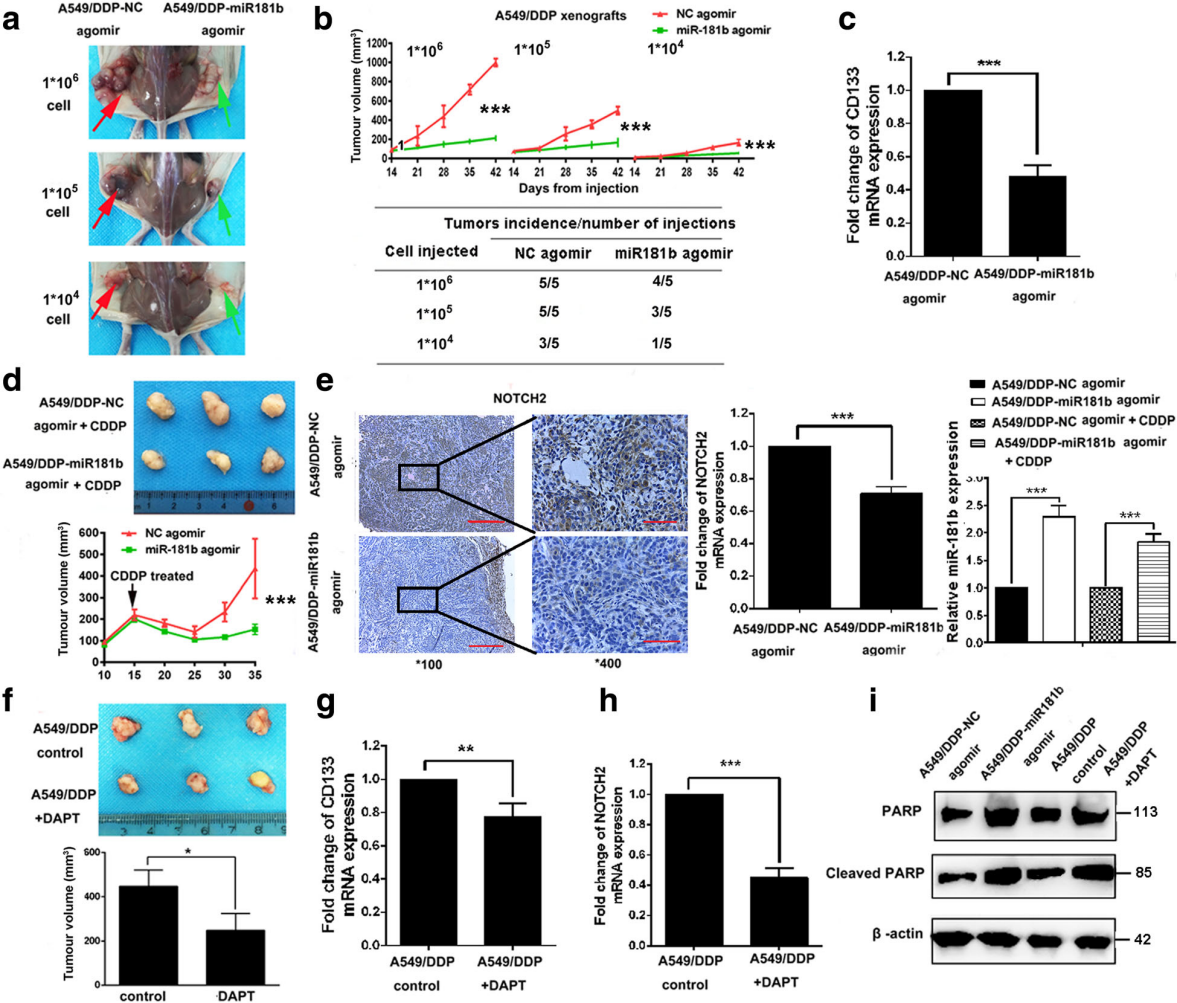
Overexpression of miR-181b inhibits CSC characteristics in vivo. Mice were treated with different doses of A549/DDP sphere cells transfected with miR-181b agomir or miR-181b-NC agomir. Representative images illustrating tumour growth (**a**) and tumour volume (**b**). **c** The mRNA level of CD133 was determined by qPCR. **d** Tumour growth and volume were evaluated after treatment with DDP (3.0 mg kg^−1^ body weight; i.p., thrice) or PBS (pH 7.4; i.p., thrice). **e** Immunostaining images showing Notch2 expression. The mRNA levels of Notch2 and miR-181b were determined by qPCR. Mice were treated with A549/DDP sphere cells and then with DAPT or the control. **f** Images show tumour growth and tumour volume. **g**, **h** The mRNA levels of CD133 and Notch2 were measured by qPCR. **i** PARP and cleaved PARP levels were also ascertained by Western blotting after the indicated treatment. Data are presented as the mean ± SD. *p < 0.05; **p < 0.01; ***p < 0.001

To determine whether the spheres have greater tumour initiation ability and to analyse the effects of miR-181b on the tumourigenicity of NSCLC cells, A549/DDP spheres at three doses (1 × 10^4^, 1 × 10^5^, and 1 × 10^6^) were inoculated subcutaneously into BALB/c nude mice (*n* = 6 mice per group). As shown in Fig. 4a and b, A549/DDP cells formed visible tumours after the injection of 1 × 10^4^ sphere cells, suggesting that sphere cells are enriched in the CSC population and have greater tumour initiation ability. The tumours treated with agomir-181b displayed imperfect tumourigenicity and slower growth, resulting in smaller tumours than those formed by agomir-NC control cells. Consistently, tumours treated with agomir-181b showed obvious downregulation of markers relevant to cancer stemness, such as CD133 (Fig. 4c), indicating that miR-181b might play a pivotal role in sphere tumourigenicity.

To evaluate whether miR-181b levels are associated with NSCLC sensitivity to DDP, mice were administered DDP treatment beginning when the mean tumour volume reached 200 mm^3^. As shown by Fig. 4d, the A549/DDP/agomir-181b group had a significantly smaller average tumour volume than did the A549/DDP/agomir-NC group after DDP treatment. To elucidate the cellular mechanisms underlying the miR-181b-mediated regulation of cancer stem cell-like properties, miR-181b and Notch2 expression was analysed in resected tissues from the treated xenograft tumours. As shown in Fig. 4e, miR-181b dramatically downregulated the expression of Notch2 in vivo. As expected, we found that inhibiting Notch signalling with the γ-secretase inhibitor DAPT also suppressed tumour growth and the expression of CD133 and Notch2 (Fig. 4f–h). Moreover, compared to the NC agomir with DDP, agomir-181b or DAPT with DDP elevated the expression of cleaved PARP (Fig. 4i). These data illustrate that the miR-181b/Notch2 axis can ameliorate chemosensitivity to DDP by regulating CSC-like activities in NSCLC cells in vivo.

### Clinical relevance of miR-181b and Notch2 in NSCLC

**Fig. 5:**
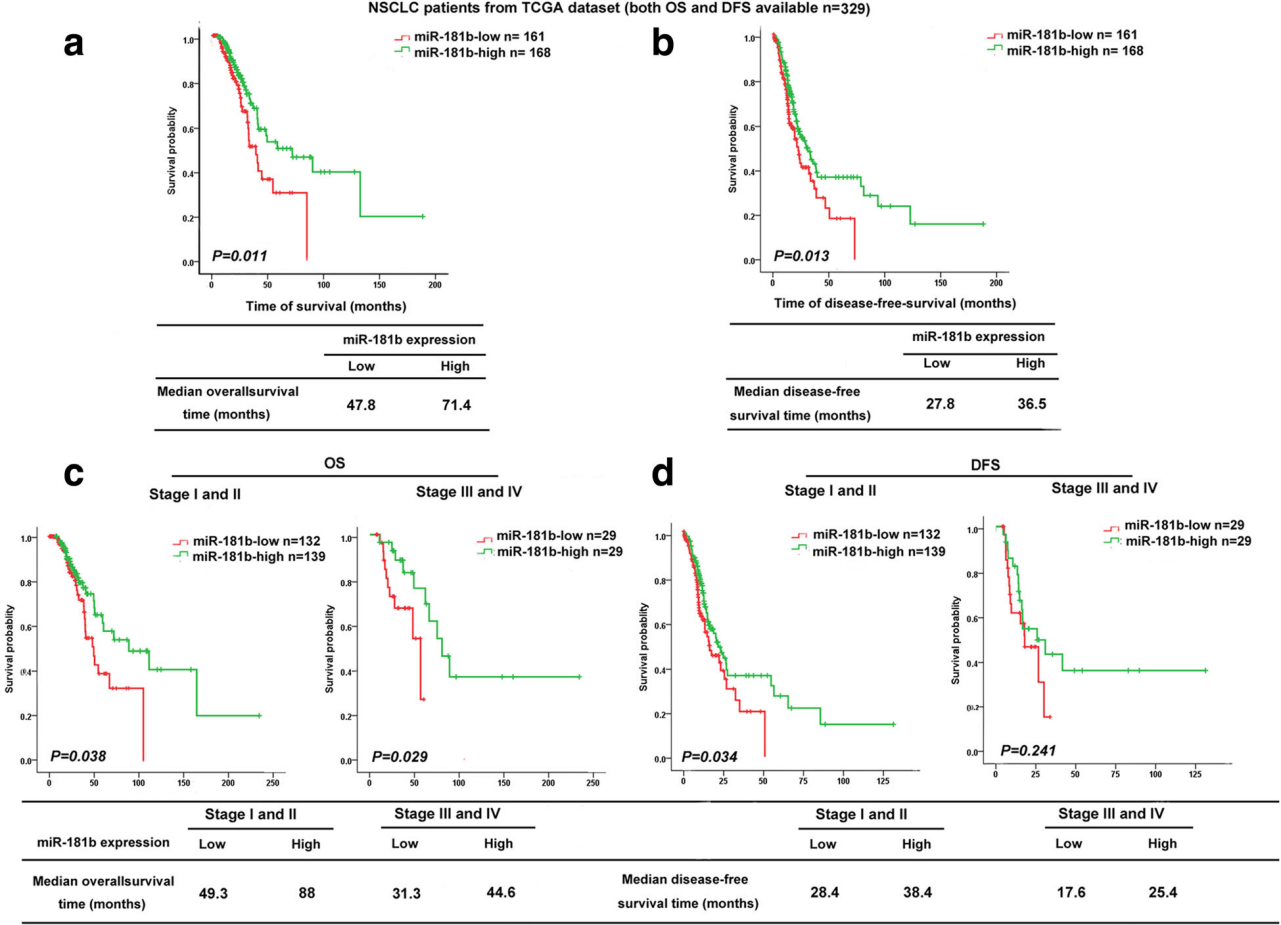
The correlation between miR-181b expression and survival time in NSCLC. Kaplan–Meier analysis of the OS (**a**) and DFS (**b**) of patients with NSCLC for whom both OS and DFS information was available in lung cancer data set from TCGA. The patients were stratified by high (greater than the median, *n* = 168) versus low (greater than or equal to the median, *n* = 161) expression of miR-181b. **c**, **d** Kaplan–Meier analysis of the correlation between miR-181b level and the OS (**c**) and DFS (**d**) of patients with NSCLC in TCGA cohort, which was further analysed for 271 NSCLC patients in stages I–II and 58 patients in stages III–IV

To determine whether miR-181b is relevant to NSCLC patient survival, miR-181b expression was analysed in NSCLC patients whose disease-free survival (DFS) and OS data were available in a lung TCGA data set (*n* = 329). The median OS for the high and low miR-181b groups was 71.4 and 47.8 months, respectively (*p* = 0.011, Fig. 5a), and the median DFS of patients with high and low miR-181b expression was 36.5 and 27.8 months, respectively (*p* = 0.013, Fig. 5b). Likewise, patients with early-stage disease (stages I and II) and late-stage disease (stages III and IV) had longer OS and DFS under conditions of high miR-181b expression than under conditions of low miR-181b expression in the TCGA lung cancer database, indicating that miR-181b levels may indicate the prognosis of NSCLC patients at different clinical stages, although there were no significant differences in DFS among patients with advanced disease (Fig. 5c, d).

**Fig. 6:**
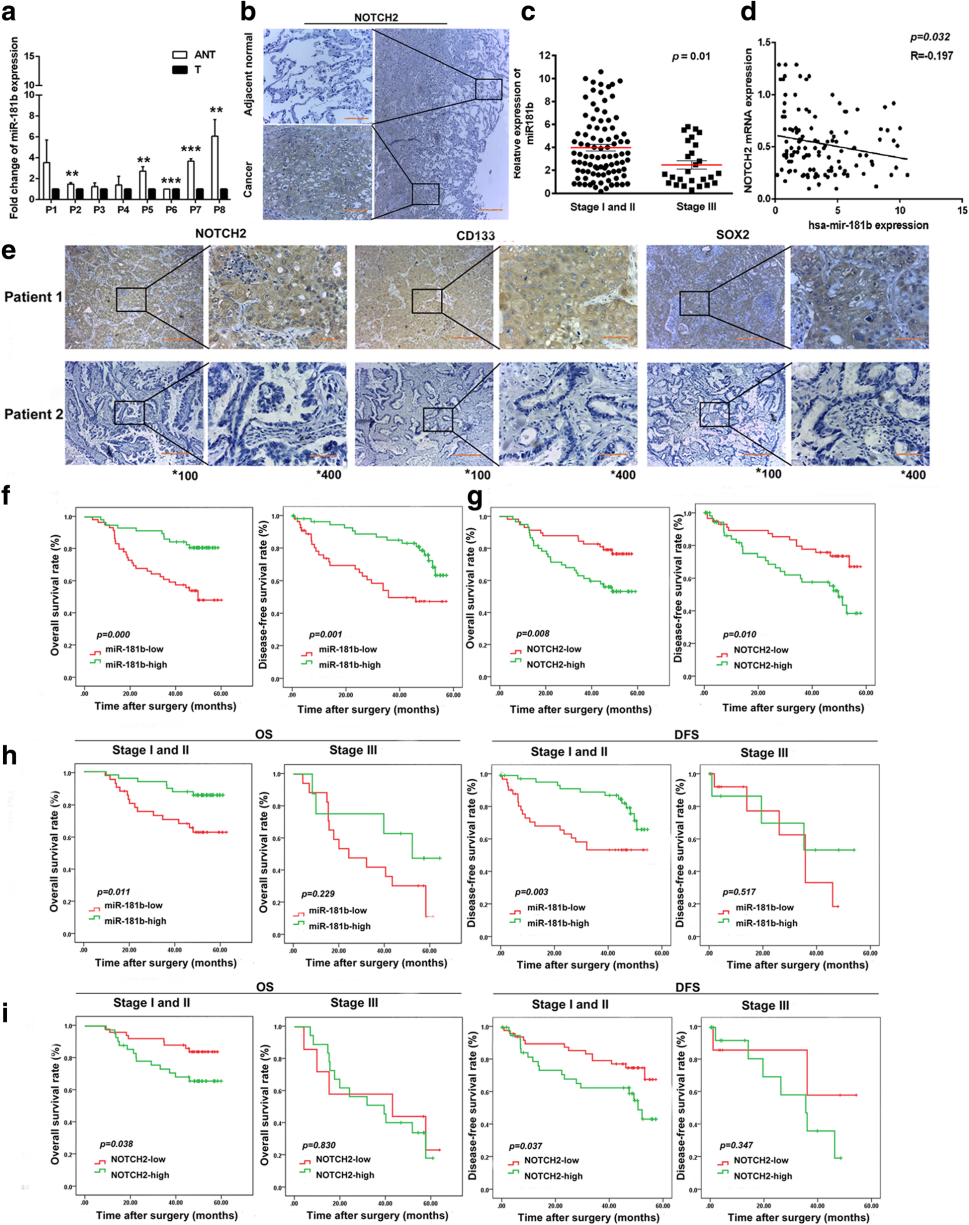
The clinical relevance of miR-181b and Notch2 in NSCLC. **a** miR-181b expression in all 8 NSCLC tumour specimens (T) compared with paired adjacent non-tumour (ANT) tissue as determined by qPCR. **b** Immunostaining images show Notch2 in tumour tissue and corresponding adjacent non-cancerous tissue. **c** miR-181b expression measured by qPCR in NSCLC patients at different disease stages. **d** A statistically significant inverse correlation between miR-181b and Notch2 mRNA levels in NSCLC tissues. **e** The expression of Notch2, CD133, and SOX2 in tumour tissues from 2 patients is shown by IHC. **f**, **g** OS and DFS curves for the high miR-181b expression group and the low miR-181b expression group, which were further analysed in different stages. **h**, **i** OS and DFS curves for the high-Notch2 expression group and the low-Notch2 expression group, which were further analysed in different stages. Data are presented as the mean ± SD. **p < 0.01; ***p < 0.001

We investigated the expression of miR-181b and Notch2 in eight pairs of human NSCLC samples and adjacent normal tissues. The expression of miR-181b was lower in eight tumour specimens (T) than in the paired adjacent non-tumour tissue (ANT) (Fig. 6a). Notch2 was mainly localized to the cytoplasm by IHC analysis. Immunostaining of Notch2 protein expression showed that tumour tissues had stronger Notch2 staining than did adjacent tissues (Fig. 6b). To investigate the expression of miR-181b/Notch2 and the correlation with stem cell-like features in NSCLC patients, the expression of miR-181b, Notch2, CD133, and SOX2 was measured in 116 patients with NSCLC from our hospital. In these 116 NSCLC patients, the level of miR-181b remained relatively high in stage I and II tumours but decreased in stage III tumours (Fig. 6c). The linear regression analysis indicated an inverse correlation between relative miR-181b and Notch2 mRNA expression in NSCLC tissues from patients (*p* = 0.032, Fig. 6d). Lower miR-181b expression was associated with a greater possibility of lymph node metastasis and a more advanced pTNM stage. Higher expression of Notch2 correlated with poor differentiation. Higher expression of CD133 was associated with poor differentiation. Higher expression of SOX2 was related to a greater possibility of lymph node metastasis (Supplementary Table S1). CD133 was mainly localized to the cytomembrane and cytoplasm, and SOX2 was mainly localized to the cytoplasm by IHC analysis (Fig. 6e). The Kaplan–Meier analysis and log-rank test confirmed the correlation of miR-181b expression with the prognosis of NSCLC patients. In Fig. 6f, g, the status of miR-181b and Notch2 was shown to be closely correlated with both DFS and OS. In addition, patients with early-stage disease (stages I and II) and high miR-181b expression had longer OS and DFS than patients with low miR-181b expression. Patients with early-stage disease (stages I and II) and low Notch2 expression had longer OS and DFS than patients with high Notch2 expression (Fig. 6h, i). Thus, these data provide evidence that miR-181b expression in tumour tissues is negatively related to Notch2 expression and the OS of patients with NSCLC. Moreover, following the evaluation of all clinicopathological factors (Table 1), multivariate analysis with the Cox proportional hazard model suggested that miR-181b status might be an independent factor predicting poor OS (HR 0.378, 95% CI 0.184–0.775; *p* = 0.008) in patients with NSCLC (Supplementary Table S2). Collectively, these results demonstrate that miR-181b/Notch2 plays an important role in stem cell-like properties and the prognosis of NSCLC.

**Table 1:** Association between miR181b expression and clinicopathological factors in patients with NSCLC

Variable	No. of patients	miR181 expression		*p* (*χ*^2^)	NOTCH2 expression				*p* (*χ*^2^)	CD133 expression				*p* (*χ*^2^)	SOX2 expression				*p* (*χ*^2^)
		High (*n*)	Low (*n*)		(−) (*n*)	(+) (*n*)	(++) (*n*)	(+++) (*n*)		(−) (*n*)	(+) (*n*)	(++) (*n*)	(+++) (*n*)		(−) (*n*)	(+) (*n*)	(++) (*n*)	(+++) (*n*)	
All cases	116	57	59		24	33	27	32		3	43	61	9		8	14	40	54	
Age (years)				0.196					0.356					0.828					0.324
≤ 55	35	14	21		8	6	10	11		1	15	17	2		2	5	8	20	
> 55	81	43	38		16	27	17	21		2	28	44	7		6	9	32	34	
Gender				0.578					0.595					0.239					0.782
Male	89	45	44		16	27	21	25		3	31	46	9		6	12	29	42	
Female	27	12	15		8	6	6	7		0	12	15	0		2	2	11	12	
Differentiation				0.684					*0.002*					*0.015*					*0.044*
Good/moderate	20	9	11		10	4	5	1		2	11	7	0		2	5	9	4	
Poor	96	48	48		14	29	22	31		1	32	54	9		6	9	31	50	
pT status				0.761					0.226					0.096					0.486
pT1	20	11	9		5	7	5	3		2	10	6	2		1	1	11	7	
pT2	84	41	43		19	20	21	24		1	27	49	7		6	12	26	40	
pT3–4	12	5	7		0	5	1	5		0	6	6	0		1	1	3	7	
pN stage				*0.005*					0.186					0.109					*0.004*
pN0	85	49	35		18	29	18	20		1	31	44	9		2	11	30	42	
pN1	14	3	11		1	3	4	6		0	5	9	0		1	2	3	8	
pN2	17	5	13		5	1	5	6		2	7	8	0		5	1	7	4	
pTNM stage				*0.022*					0.186					0.055					0.131
I	65	34	31		13	23	15	14		1	23	32	9		2	8	25	30	
II	26	7	19		6	8	4	8		0	9	17	0		1	4	6	15	
III	25	16	9		5	2	8	10		2	11	12	0		5	2	9	9	

## Discussion

Chemotherapy resistance is a major obstacle to the clinical treatment of NSCLC. During the past decade, CSCs have become increasingly established in many malignancies and have been shown to play significant roles in tumourigenesis and tumour recurrence, which are considered the roots of drug resistance [18, 37]. Targeting CSCs has some advantages in eliminating the underlying cause of tumours and reducing side effects. CSCs possess many features of embryonic or tissue stem cells, and one or more highly conserved signal transduction pathways involved in development and tissue homeostasis, such as the Notch, Hedgehog, and Wnt pathways, are persistently activated in CSCs [19, 20, 40]. Here, we provide the first evidence that miR-181b suppresses stemness by targeting Notch2 signalling, and we find that the miR-181b-Notch2 axis may play a crucial role in chemoresistance and be a therapeutic target in NSCLC.

CSC-like properties have been shown to be regulated by miRNAs in various cancer types. Loss of miR-204 expression enhances glioma migration and the stem cell-like phenotype. miR-142-5p induces CSC-like properties in cutaneous squamous cell carcinoma via inhibiting PTEN [21]. miR-708-5p was found to inhibit lung cancer stem cell-like phenotypes through repressing Wnt/β-catenin signalling [22]. However, the functional role of miR-181b in NSCLC, especially in regulating cancer stem cell-like properties, remains largely unknown [38, 39].

In our earlier study, we proved that miR-181b inhibits cell proliferation, increases chemosensitivity to DDP, and suppresses migration and invasion in NSCLC. In this study, miR-181b was found to be an important player in regulating stem cell-like properties of NSCLC. Overexpression of miR-181b can suppress stem-like characteristics, reduce DDP resistance, and promote the apoptosis of NSCLC in vitro. In vivo, agomir-181b significantly increased chemosensitivity to DDP by regulating CSC-like activities. Furthermore, the status of miR-181b and Notch2 was shown to be closely correlated with both DFS and OS.

Accumulating evidence demonstrates that Notch signalling can promote the proliferation of tumour cells and enhance CSC properties and is implicated in several cancers that exhibit resistance to conventional chemotherapy [23–25]. There are four receptors in the mammalian Notch family (Notch1–4), and they are stimulated via binding to the corresponding ligands. The intracellular domain of Notch (NICD) is exposed after the activation of Notch signalling, and the NICD cleavage products subsequently translocate to the nucleus. Then, the Notch target genes Hes-1 and Hey-1 are activated after interacting with a transcription complex [26]. In ovarian cancer, miR-136 suppresses cancer stem cell properties and magnifies the antitumour effects of paclitaxel against chemoresistance by targeting Notch3 [27]. CD44^+^/CD133^+^-associated multidrug resistance is reversed by miR-139-5p, with a reduction in Notch1 in colorectal carcinoma cells [28]. Studies have shown that Notch2 is regulated by miR-107 in glioma [29]. By suppressing Notch2 signalling, c8orf4 negatively regulates the self-renewal of cancer stem cells in the liver [30]. In neoadjuvant-treated gastric cancer, the expression profiling of stem cell-related genes indicated that Notch2, GSK3β, and β-catenin gene signatures predict survival [31]. Targeting CSCs with inhibitors of Notch signalling promotes cell differentiation, increases sensitivity to chemotherapy, and reduces metastasis [32]. In NSCLC, Notch signalling combined with several transcriptional factors exerts an important influence on the initiation of lung cancer and contributes to NSCLC progression. In advanced NSCLC, the activities of Notch1 and Notch3 are higher and are associated with poor prognosis. Increased expression of Notch2 was found by RT-PCR in patients with lung adenocarcinoma compared to that in other histology types [33]. Konishi et al. reported that in a number of lung cancer cell lines, the Jagged 1 and Notch transcriptional target genes Hey1 and HES1 are elevated. Blocking this signalling pathway with MRK-003, a γ-secretase inhibitor, downregulated Notch3 signalling, restrained cancer cell growth, and promoted tumour cell apoptosis in vitro and in vivo [26]. Hassan et al. confirmed Notch activity as a marker of CSCs in NSCLC. A Notch GFP-reporter construct was used to identify cells with high Notch activity, which were found to have an enhanced capacity to form tumourspheres and to be more resistant to cisplatin and docetaxel chemotherapy [34]. In a more recent study by Yen et al., a novel cross-reactive antibody, OMP-59R5, that selectively blocks the function of both Notch2 and Notch3 was assessed for its antitumour effects in patient-derived xenograft (PDX) models [35]. In our study, we found that miR-181b has the potential to suppress Notch2 expression by binding directly to its 3′-UTR. In addition, silencing Notch2 strikingly attenuated stem cell-like traits, such as sphere formation, the CD133^+^ population, and the expression of stemness-related transcription factors. We also showed that the miR-181b mimic markedly downregulated Notch2, NICD2, HES1, and HEY1 expression, suggesting that Notch2 plays a vital role in miR-181b-mediated stemness of NSCLC cells. Likewise, miR-181b was reduced in the patient samples and was inversely correlated with Notch2 expression. Our results show that the miR-181b/Notch2 signalling pathway has a significant effect on stem cell-like properties and may perform a novel regulatory function in drug resistance in NSCLC.

miR-181b is thought to have important effects on apoptosis, metastasis, and chemoresistance [41, 42]. Previously, we demonstrated that miR-181b acts as a tumour suppressor and enhances chemosensitivity to DDP in NSCLC. This work further explored the mechanism by which downregulated miR-181b results in elevated Notch2 expression and promotes stem cell-like properties, thereby contributing to chemoresistance. In addition, miR-181b was associated with patient outcome and showed potential utility as a prognostic indicator in NSCLC patients (Table 2). miR-181b-mediated replacement therapy may therefore represent a promising compensator in chemoresistance, and modulating the miR-181b/Notch2 signalling pathway may be an efficient therapeutic strategy for lung cancer. Large-scale and prospective clinical trials are needed to further investigate whether and how miR-181b and other miRNAs can be used alone or in combination in the clinic.

**Table 2:** Prognostic factors for lung SCC patients

Variables	HR	Univariate 95% CI	*p*	HR	Multivariate 95% CI	*p*
OS						
Age (years)						
≤ 55 vs. > 55	1.002	0.965–1.039	0.932			
Gender						
Male vs. female	0.309	0.110–1.868	0.076			
Differentiation						
Good/moderate vs. poor	0.717	0.330–1.558	0.401			
pT status						
pT2–4 vs. pT1	3.747	1.721–8.160	*0.001*			0.580
pN stage						
pN1–2 vs. pN0	3.090	1.569–6.086	*0.001*			0.180
pTNM stage						
II–III vs. I	4.921	2.629–9.210	0.000	5.744	1.908–17.290	*0.002*
miR181b expression						
Low vs. high	0.293	0.147–0.586	0.001	0.378	0.184–0.775	*0.008*
NOTCH2 expression						
Low vs. high	1.436	1.062–1.941	*0.019*			0.242
CD133 expression						
Low vs. high	1.229	0.776–1.945	0.380			
SOX2 expression						
Low vs. high	1.013	0.718–1.430	0.939			

*CI* confidence interval, *HR* hazard ratio, *OS* overall survival

## Conclusions

Ectopic miR-181b expression suppressed cancer stem cell properties and enhanced the sensitivity to DDP treatment by directly targeting Notch2. Decreased miR-181b expression and increased Notch2 expression were observed to have a significant relationship with OS and CSC-like properties in NSCLC patients. Our results suggest that the miR-181b-Notch2 axis might be a potential target for the treatment of chemoresistance in NSCLC.

## Additional files

Additional file 1: **Figure S1.** Increased miR-181b suppresses CSC properties in NSCLC. (A) The miR-181b expression in A549/DDP, A549, H1650, H460 and HBE normal lung epithelial cells was measured by qPCR. (B) H1650 and H460 cells were transfected with miR-181b mimics, miR-181b inhibitors or the control. The number of tumourspheres was counted, and the morphology was observed under a light microscope. (C) CD133^+^ H1650 and H460 cells were analysed by flow cytometry. (D) The mRNA levels of KLF4, SOX2, NANOG, CD133 and ALDH were measured by qPCR. (E) A549 and H1650 cells were treated with miR-181b inhibitors, and A549/DDP and H460 cells were treated with miR-181b mimics. The miR-181b expression in each group was determined by qPCR. Bars represent 200 μm for low-power lens and 50 μm for high-power lens. Data are presented as the mean ± SD. * *p* < 0.05; ** *p* < 0.01; *** *p* < 0.001. (TIF 1468 kb)

Additional file 2 **Figure S2.** Restoration of miR-181b increases the chemosensitivity of NSCLC cells to DDP. H1650 and H460 cells were transfected with miR-181b mimics, miR-181b inhibitors or the control. (A, B) IC50 values were measured by CCK analysis with different concentrations of cisplatin. (C) The apoptotic percentage was determined by flow cytometry. (C) Western blotting showed Bcl-2 and cleaved caspase-3 expression levels. Data are presented as the mean ± SD. * *p* < 0.05; ** *p* < 0.01; *** *p* < 0.001. (TIF 788 kb)

## Abbreviations

CSC: Cancer stem cell
DDP: Cisplatin
miR-181b: MicroRNA-181b
miRNAs: MicroRNAs
NSCLC: Non-small cell lung cancer

## References

[1] DeSantis CE, Lin C, Mariotto AB (2014). Cancer treatment and survivorship statistics. CA Cancer J Clin.

[2] Fennell DA, Summers Y, Cadranel J (2016). Cisplatin in the modern era: the backbone of first-line chemotherapy for non-small cell lung cancer. Cancer Tre Rev.

[3] Allen KE, Weiss GJ (2010). Resistance may not be futile: microRNA biomarkers for chemoresistance and potential therapeutics. Mol Cancer Ther.

[4] Sarkar FH, Li Y, Wang Z (2010). Implication of microRNAs in drug resistance for designing novel cancer therapy. Drug Resis Updat.

[5] Szakacs G, Paterson JK, Ludwig JA (2006). Targeting multidrug resistance in cancer. Nat Rev Drug Discov.

[6] Nguyen LV, Vanner R, Dirks P (2012). Cancer stem cells: an evolving concept. Nat Rev Cancer.

[7] Bitarte N, Bandres E, Boni V (2001). MicroRNA-451 is involved in the self-renewal, tumorigenicity, and chemoresistance of colorectal cancer stem cells. Stem Cells.

[8] Park EY, Chang E, Lee EJ (2014). Targeting of miR34a-NOTCH1 axis reduced breast cancer stemness and chemoresistance. Cancer Res.

[9] Shi ZM, Wang L, Shen H (2017). Downregulation of miR-218 contributes to epithelial-mesenchymal transition and tumor metastasis in lung cancer by targeting Slug/ZEB2 signaling. Oncogene.

[10] Yang J, Liu H, Wang H (2013). Down-regulation of microRNA-181b is a potential prognostic marker of non-small cell lung cancer. Pathol Res Pract.

[11] Wang X, Chen X, Meng Q (2015). MiR-181b regulates cisplatin chemosensitivity and metastasis by targeting TGFbetaR1/Smad signaling pathway in NSCLC. Sci Rep.

[12] Zhu W, Shan X, Wang T (2010). miR-181b modulates multidrug resistance by targeting BCL2 in human cancer cell lines. Int J Cancer.

[13] Li P, Lu X, Wang Y (2010). MiR-181b suppresses proliferation of and reduces chemoresistance to temozolomide in U87 glioma stem cells. J Bio Res.

[14] Eramo A, Lotti F, Sette G (2008). Identification and expansion of the tumorigenic lung cancer stem cell population. Cell Death Differ.

[15] Tirino V, Camerlingo R, Franco R (2009). The role of CD133 in the identification and characterisation of tumour-initiating cells in non-small-cell lung cancer. Eur J Cardiothorac Surg.

[16] Dean M, Fojo T, Bates S (2005). Tumour stem cells and drug resistance. Nat Rev Cancer.

[17] DeSano JT, Xu L (2009). MicroRNA regulation of cancer stem cells and therapeutic implications. AAPS J.

[18] Reya T, Morrison SJ, Clarke MF (2001). Stem cells, cancer, and cancer stem cells. Nature.

[19] MacDonagh L, Gray SG, Breen E (2016). Lung cancer stem cells: the root of resistance. Cancer Let.

[20] Takebe N, Miele L, Harris PJ (2015). Targeting Notch, Hedgehog, and Wnt pathways in cancer stem cells: clinical update. Nat Rev Clin Oncol.

[21] Bai X, Zhou Y, Yang M (2018). MicroRNA-142-5p induces cancer stem cell-like properties of cutaneous squamous cell carcinoma via inhibiting PTEN. J Cell Biochem.

[22] Hu X, Liu T, Wu X (2018). Downregulation of DNMT3A by miR-708-5p inhibits lung cancer stem cell-like phenotypes through repressing Wnt/beta-catenin signaling. Clin Cancer Res.

[23] Androutsellis-Theotokis A, Leker RR, Soldner F (2006). Notch signalling regulates stem cell numbers in vitro and in vivo. Nature.

[24] Croker AK, Allan AL (2008). Cancer stem cells: implications for the progression and treatment of metastatic disease. J Cellular Mol Med.

[25] Mullendore ME, Koorstra JB, Li YM (2009). Ligand-dependent Notch signaling is involved in tumor initiation and tumor maintenance in pancreatic cancer. Clin Cancer Res.

[26] Konishi J, Kawaguchi KS, Vo H (2007). Gamma-secretase inhibitor prevents Notch3 activation and reduces proliferation in human lung cancers. Cancer Res.

[27] Jeong JY, Kang H, Kim TH (2017). MicroRNA-136 inhibits cancer stem cell activity and enhances the anti-tumor effect of paclitaxel against chemoresistant ovarian cancer cells by targeting Notch3. Cancer Lett.

[28] Xu K, Shen K, Liang X (2016). MiR-139-5p reverses CD44+/CD133+-associated multidrug resistance by downregulating NOTCH1 in colorectal carcinoma cells. Oncotarget.

[29] Chen L, Chen XR, Zhang R (2013). MicroRNA-107 inhibits glioma cell migration and invasion by modulating Notch2 expression. J Neuro-Oncol.

[30] Zhu P, Wang Y, Du Y (2015). C8orf4 negatively regulates self-renewal of liver cancer stem cells via suppression of NOTCH2 signalling. Nat Commun.

[31] Bauer L, Langer R, Becker K (2012). Expression profiling of stem cell-related genes in neoadjuvant-treated gastric cancer: a NOTCH2, GSK3B and beta-catenin gene signature predicts survival. PLoS One.

[32] Liu J, Mao Z, Huang J (2014). Blocking the NOTCH pathway can inhibit the growth of CD133-positive A549 cells and sensitize to chemotherapy. Biochem Biophys Res Commun.

[33] Chen CY, Chen YY, Hsieh MS (2017). Expression of Notch gene and its impact on survival of patients with resectable non-small cell lung cancer. J Cancer.

[34] Hassan KA, Wang L, Korkaya H (2013). Notch pathway activity identifies cells with cancer stem cell-like properties and correlates with worse survival in lung adenocarcinoma. Clin Cancer Res.

[35] Yen WC, Fischer MM, Axelrod F (2015). Targeting Notch signaling with a Notch2/Notch3 antagonist (tarextumab) inhibits tumor growth and decreases tumor-initiating cell frequency. Clin Cancer Res.

[36] Wang X, Chen X, Meng Q (2015). MiR-181b regulates cisplatin chemosensitivity and metastasis by targeting TGFβR1/Smad signaling pathway in NSCLC. Sci Rep.

[37] Papaccio F, Paino F, Regad T (2017). Concise review: cancer cells, cancer stem cells, and mesenchymal stem cells: influence in cancer development. Stem Cells Transl Med.

[38] Fasano M, Della Corte CM, Capuano A (2015). A multicenter, open-label phase II study of metformin with erlotinib in second-line therapy of stage IV non-small-cell lung cancer patients: treatment rationale and protocol dynamics of the METAL trial. Clin Lung Cancer.

[39] Shi Q, Zhou Z, Ye N (2017). MiR-181a inhibits non-small cell lung cancer cell proliferation by targeting CDK1. Cancer Biomark.

[40] Pirozzi G, Tirino V, Camerlingo R (2013). Prognostic value of cancer stem cells, epithelial-mesenchymal transition and circulating tumor cells in lung cancer. Oncol Rep.

[41] Huang P, Ye B, Yang Y, Shi J, Zhao H (2015). MicroRNA-181 functions as a tumor suppressor in non-small cell lung cancer (NSCLC) by targeting Bcl-2. Tumour Biol.

[42] Caputi M, De Luca L, Papaccio G (1997). Prognostic role of cyclin D1 in non small cell lung cancer: an immunohistochemical analysis. Eur J Histochem.

