# Supplementary figures and images for: miR-181b/Notch2 overcome chemoresistance by regulating cancer stem cell-like properties in NSCLC
Source: Stem Cell Res Ther. 2018 Nov 23;9:327. doi: 10.1186/s13287-018-1072-1 (PMC6260863; doi:10.1186/s13287-018-1072-1)

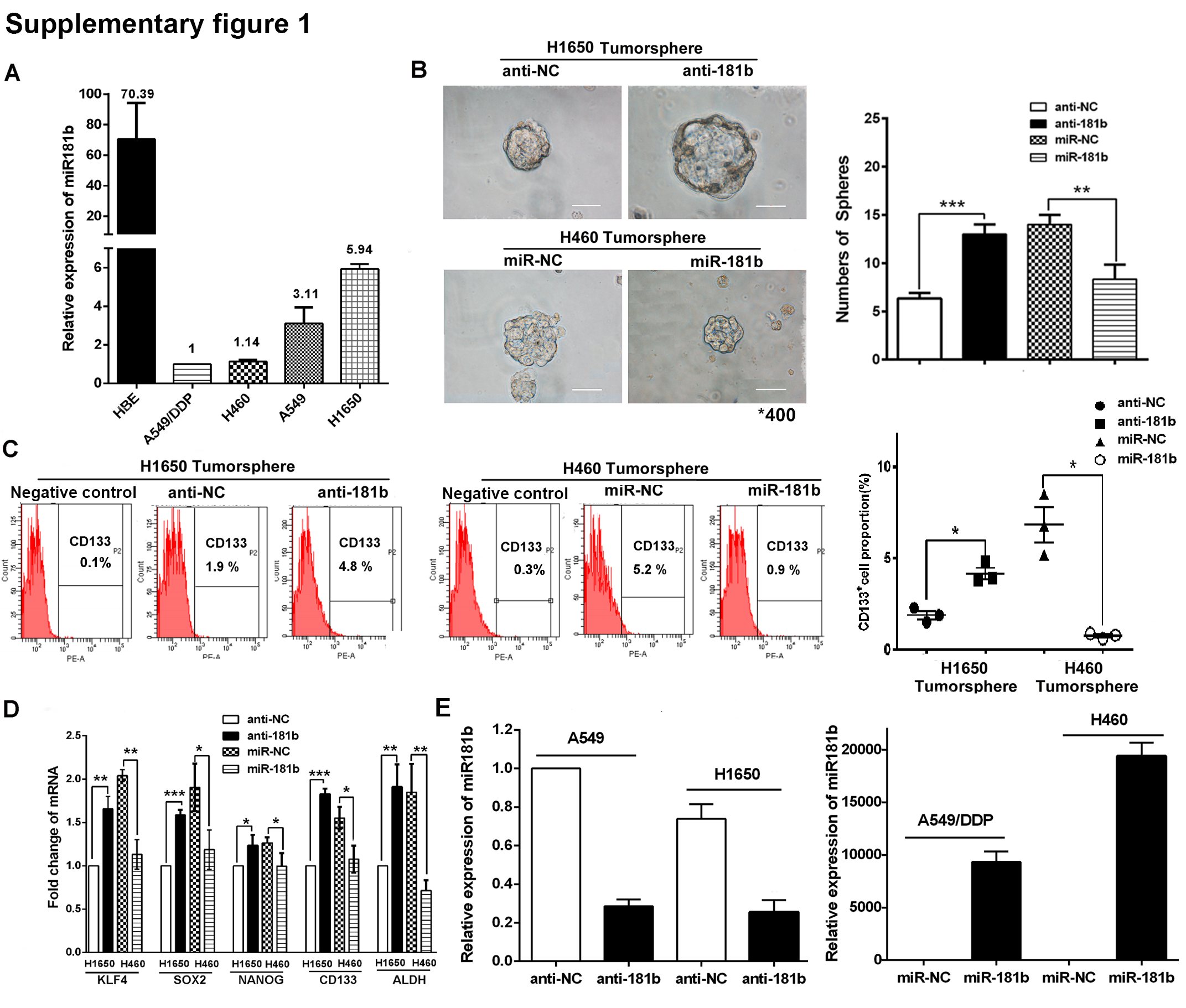

Supplement: Supplementary file 1 — Figure S1. Increased miR-181b suppresses CSC properties in NSCLC. (A) The miR-181b expression in A549/DDP, A549, H1650, H460 and HBE normal lung epithelial cells was measured by qPCR. (B) H1650 and H460 cells were transfected with miR-181b mimics, miR-181b inhibitors or the control. The number of tumourspheres was counted, and the morphology was observed under a light microscope. (C) CD133+ H1650 and H460 cells were analysed by flow cytometry. (D) The mRNA levels of KLF4, SOX2, NANOG, CD133 and ALDH were measured by qPCR. (E) A549 and H1650 cells were treated with miR-181b inhibitors, and A549/DDP and H460 cells were treated with miR-181b mimics. The miR-181b expression in each group was determined by qPCR. Bars represent 200 μm for low-power lens and 50 μm for high-power lens. Data are presented as the mean ± SD. * p < 0.05; ** p < 0.01; *** p < 0.001. (TIF 1468 kb) [file 13287_2018_1072_MOESM1_ESM.tif]

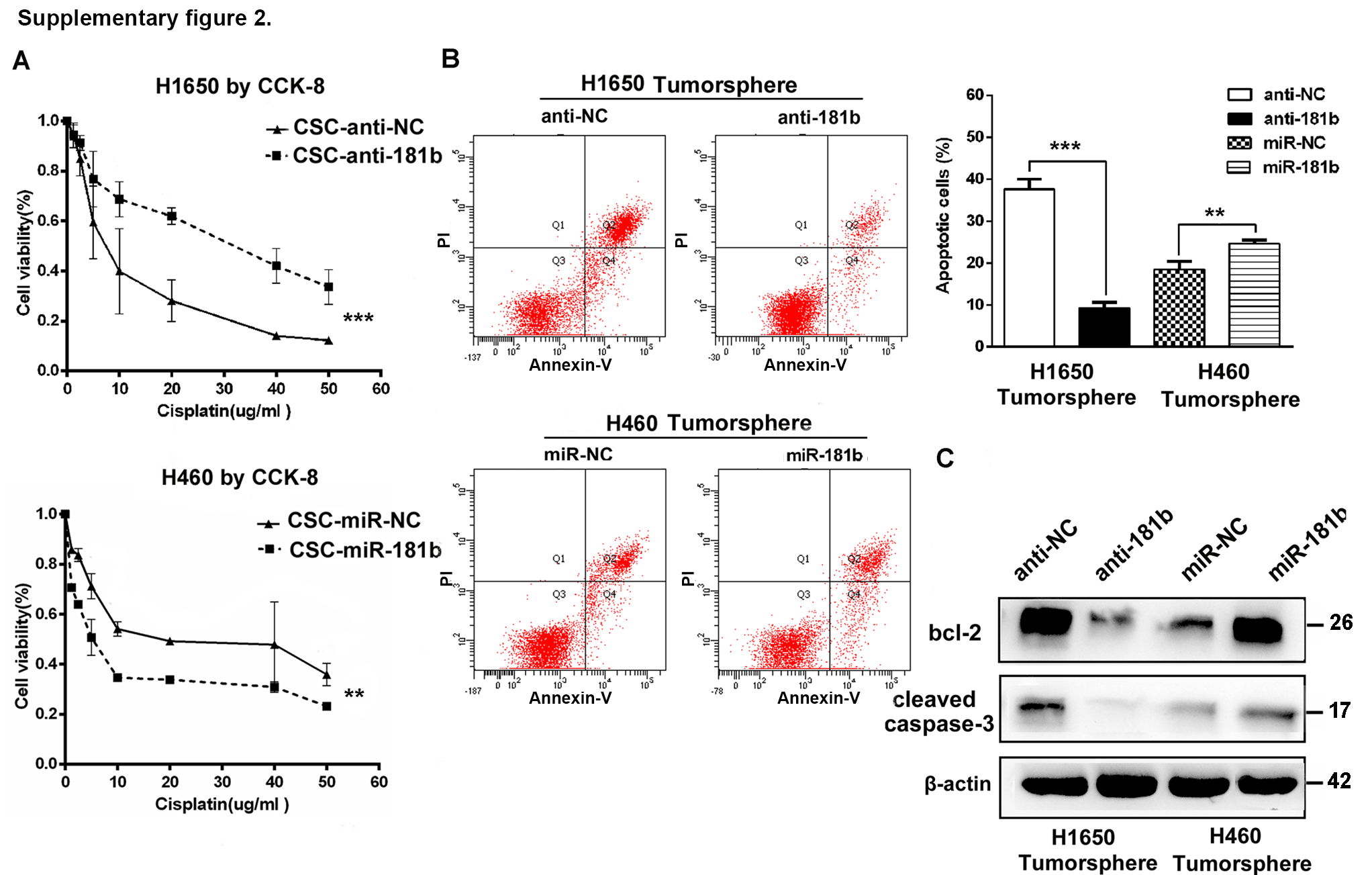

Supplement: Supplementary file 2 — Figure S2. Restoration of miR-181b increases the chemosensitivity of NSCLC cells to DDP. H1650 and H460 cells were transfected with miR-181b mimics, miR-181b inhibitors or the control. (A, B) IC50 values were measured by CCK analysis with different concentrations of cisplatin. (C) The apoptotic percentage was determined by flow cytometry. (C) Western blotting showed Bcl-2 and cleaved caspase-3 expression levels. Data are presented as the mean ± SD. * p < 0.05; ** p < 0.01; *** p < 0.001. (TIF 788 kb) [file 13287_2018_1072_MOESM2_ESM.tif]
